# Corncob and sugar beet pulp induce specific sets of lignocellulolytic enzymes in *Penicillium purpurogenum*

**DOI:** 10.1080/21501203.2018.1517830

**Published:** 2018-09-11

**Authors:** Wladimir Mardones, Eduardo Callegari, Jaime Eyzaguirre

**Affiliations:** a Facultad de Ciencias Biológicas, Universidad Andrés Bello, Santiago, Chile; b BRIN-USDSSOM Proteomics Facility, University of South Dakota, Vermillion, SD, USA

**Keywords:** Secretome, lignocellulose biodegradation, mass spectrometry analysis, CAZymes, *Penicillium purpurogenum*

## Abstract

*Penicillium purpurogenum* is a filamentous fungus, which grows on a variety of natural carbon sources and secretes a large number of enzymes involved in cellulose, hemicelluloses and pectin biodegradation. The purpose of this work has been to identify potential lignocellulolytic enzymes and to compare the secreted enzymes produced when the fungus is grown on sugar beet pulp (rich in cellulose and pectin) and corn cob (rich in cellulose and xylan). Culture supernatants were subjected to two-dimensional nano-liquid chromatography/tandem mass spectrometry. Using MASCOT and a genome-derived protein database, the proteins present in the supernatant were identified. The putative function in the degradation of the polysaccharides was determined using dbCAN software. The results show that there is a good correlation between the polysaccharide composition of the carbon sources and the function of the secreted enzymes: both cultures are rich in cellulases, while sugar beet pulp induces pectinases and corncob, xylanases. The eventual biochemical characterisation of these enzymes will be of value for a better understanding of the biodegradation process performed by the fungus and increase the availability of enzymes for biotechnological methods associated with this process.

## 1. Introduction

Lignocellulose, the most abundant renewable resource in the biosphere, is a valuable raw material for an increasing number of biotechnological applications such as bioethanol production, and an important source of chemical products (Ragauskas et al. 2006). It is composed of a polyphenol (lignin) and a variety of polysaccharides, the most important being cellulose, hemicelluloses and pectin (Anwar et al. 2014).

The utilisation of the lignocellulose polysaccharides (particularly for bioethanol production) requires their degradation (or saccharification) to their component monosaccharides. This can be achieved by chemical means (which leads to the generation of undesirable products and environmental contamination) or by utilising enzymes, which can operate at milder conditions and avoid the formation of side-products (Blanch 2012).

Cellulose is composed solely of glucose moieties linked by β (1→4) bonds and for its biodegradation utilises a set of three enzymes: endoglucanases, exoglucanases and β-glucosidases (Beguin and Aubert 1994). Of the hemicelluloses, the most abundant is xylan, a heteropolysaccharide with a main-chain of xylose residues linked β (1→4) and bound to several types of substituents (arabinose, methyl glucuronic acid and cinnamic acids). It involves for its saccharification a more complex set of enzymes (Perez et al. 2002). Finally, pectin, the most heterogeneous of these polysaccharide, requires an even more elaborate group of enzymes, many of them still unknown (Jayani et al. 2005).

Cocktails of enzymes are necessary for the effective degradation of lignocellulose from different sources and composition (Mohanram et al. 2013); thus a knowledge of these enzymes (listed in the Carbohydrate Active Enzymes database, CAZy (Cantarel et al. 2009)) and their properties is important. These enzymes are produced mainly by bacteria (Lo et al. 2009) and fungi (Persson et al. 1991). Fungi are the preferred source because some of them are very active protein secretors. Of particular interests are strains of *Trichoderma, Aspergillus* and *Penicillium* (Persson et al. 1991).

Our laboratory has used as a model for the study of lignocellulolytic enzymes a locally isolated strain of *Penicillium* (Musalem et al. 1984), which has been registered at the ATCC as MYA-38. This soft-rot fungus grows on a variety of lignocellulolytic natural carbon sources (i.e. sugar beet pulp, corncob, etc.) (Steiner et al. 1994; González-Vogel et al. 2011). It secretes to the medium a large number of cellulose-, xylan- and pectic-degrading enzymes, some of which have been characterised and sequenced (Chavez et al. 2006; Ravanal et al. 2010). Recently, a sequence of its genome has been obtained (Mardones et al. 2018), and a high number of genes coding for possible lignocellulolytic enzymes have been identified.

The purpose of this work has been: (1) To identify in the secretome potential lignocellulose-degrading enzymes whose genes are present in the genome. (2) To compare the production of potential enzymes when the fungus is grown on two carbon sources of different composition (sugar beet pulp and corncob). (3) To compare the results obtained in this work using two-dimensional nano-liquid chromatography/tandem mass spectrometry for identification of potential enzymes with the method used by Navarrete et al. (2012) (2-D electrophoresis and mass spectrometry).

## 2. Materials and methods

### 2.1. Fungal strain and culture conditions

*Penicillium purpurogenum* ATCC strain MYA-38 was grown in Mandel’s medium as described previously (Hidalgo et al. 1992). Liquid cultures were incubated for 4 days at 28°C in an orbital shaker (200 rpm) using 1% corncob or sugar beet pulp as carbon sources. The incubation time was chosen in order to compare results with previous work by Navarrete et al. (2012). The supernatants of these cultures were utilised for secretome analysis. One biological sample was used per carbon source.

### 2.2. Secreted lignocellulolytic enzymes identification

To identify the enzymes secreted by *P. purpurogenum*, two-dimensional nano-liquid chromatography/tandem mass spectrometry (2D-nano-LC-MS/MS) was utilised. The four-day fungal cultures were filtered and concentrated by means of ultrafiltration using a 10 kDa cut-off Centricon (Amicon, MA, USA). Four volumes of de-ionised water were added and concentrated anew. The proteins were reduced in solution with 50 mM DTT (Sigma, MO, USA) at 65°C for 5 min, alkylated with 100 mM iodoacetamide (Sigma) and digested with sequencing grade trypsin (Promega, WI, USA) overnight at 37°C. The digestion was stopped by the addition of 0.5% acetic acid, frozen in dry ice and concentrated on a SpeedVac centrifuge (Thermo Fisher Scientific, MA, USA). The trypsin-digested peptides were dissolved in 100 mM ammonium formate pH 10 and separated through 2D-nano-LC with dilution using a 2D-nanoAcquity UPLC (Waters, MA, USA). The first dimension was performed in a XBridge BEH130 C18, 5 µm, 300 µm × 50 mm NanoEase Column (Waters) using solvent A1:20 mM ammonium formate pH 10 and B1:100% acetonitrile (Fisher Optima, LC-MS grade). The flow rate was 2 µL/min, and 10 different step gradients (dilution method) were performed separately for 20 min. The second dimension included trapping and desalting online through a 180 µm × 20 mm, 5 µm Symmetry C18 nanoAcquity UPLC trap column (Waters) at a flow rate of 20 µL/min, with a 99% A2 (0.1% formic acid in water) and 1% B2 (100% acetonitrile, 0.1% formic acid) for 20 min. After the peptides were desalted and concentrated, they were separated online in the second dimension through a BEH130C18 1.7 µm, 100 µm × 100 mm nanoAcquity UPLC column. The standard solvent gradient used was: 0–2 min, 3% B2 isocratic; 2–40 min, 3–85% B2 linear, at a flow rate of 400 nL/min for 60 min (for details see: Dong et al. 2012; Callegari 2016). The eluted ions were analysed by one full precursor MS scan (400–1500 m/z) followed by four MS/MS scans of the most abundant ions detected in the precursor MS scan while operating under dynamic exclusion or direct data acquisition system. Spectra obtained in the positive ion mode with nano ESI-Q-Tof Synapt G1 mass spectrometer (Waters) were deconvoluted and analysed using the MassLynx software 4.1 (Waters). A peak list (PKL format) was generated to identify +1 or multiple charged precursor ions from the mass spectrometry data file. The instrument was calibrated in MS/MS mode using 100 fmole of human (Glu1)-Fibrinopeptide B (Sigma) with a RMS residual of 3.857 e-4 amu or 6.9413 e-1 ppm. Parent mass (MS) and fragment mass (MS/MS) peak ranges were 400–2000 Da and 65–2000 Da, respectively.

### 2.3. Database search

Mascot server v2.5 and Mascot Daemon Toolbox v2.5 (www.matrix-science.com) in MS/MS ion search mode (local licenses) were applied to conduct peptide matches (peptide masses and sequence tags) and protein searches. These searches were performed against the *P. purpurogenum* all proteins database (11,555 sequences; 592,686 residues) and the NCBInr database (all entries) 20,160,830 (93,482,448 sequences; 34,454,162,062 residues) and the taxonomy filter for fungi (5,915,770 sequences) (1 March 2018). The *P. purpurogenum* genome assembly (Bioproject PRJNA276974, NCBI) and protein sequences (http://ppurdb.cmm.uchile.cl/material) are publicly available (see Mardones et al. 2018). The following parameters were set for the search: carbamidomethyl (C) on cysteine was set as fixed; variable modifications included asparagine and glutamine deamidation and methionine oxidation, as well as Error Tolerance mode. One missed cleavage was allowed for regular searching; monoisotopic masses were counted; the precursor peptide mass tolerance was set at 2 Da; fragment mass tolerance was 0.3 Da, and the ion score or expected cut-off was set at 5. Known keratin contaminant ions (keratin) were excluded. The MS/MS spectra were searched with MASCOT using a 95% confidence interval (C.I. %) threshold (*p* < 0.05), and a minimum score of 34 was used for peptide identification. All proteins identified were found in these domains.

### 2.4. Bioinformatics analysis

dbCAN (http://csbl.bmb.uga.edu/dbCAN/) software was used to predict enzymatic function using the CAZy database families (http://www.cazy.org/). Protein sequence searches were performed with DELTA Blast (https://www.ncbi.nlm.nih.gov/books/NBK279685/).

## 3. Results and discussion

**Figure 1.**
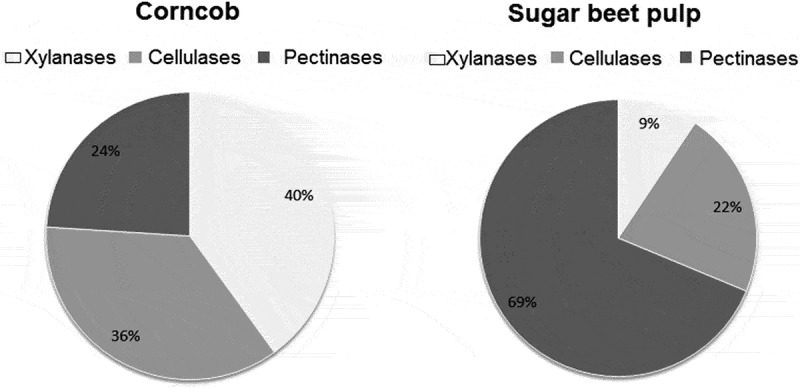
Per cent distribution of the number of lignocellulose-degrading enzymes in the secretomes of *Penicillium purpurogenum* grown on sugar beet pulp or corncob.

Supernatants of cultures grown on sugar beet pulp and corncob as carbon sources were subjected to 2D-nano-LC-MS/MS as described in Materials and methods. The purpose of this analysis was to compare the presence of CAZymes secreted in cultures using the two different carbon sources. Sugar beet pulp is rich in cellulose (about 20%) but its main component is pectin (about 50%), while xylose constitutes only 1.7% and lignin less than 1% (Saulnier and Thibault 1999). Corncob, on the other hand, contains (dry weight) 35% cellulose, 35% xylan, 14% lignin and 3% arabinan (Yang et al. 2006). Thus, one would expect to have cellulases induced under both conditions, and pectinolytic enzymes in preference in the sugar beet pulp medium and xylanases in the corncob culture. Figures S1 and S2 show the MASCOT search results of the raw data of the corncob and sugar beet pulp secretome analysis, respectively. Table S1 presents the CAZymes found in the sugar beet pulp culture secretome with their respective e-values assigned by dbCAN. A total of 42 CAZymes were identified; 6 correspond to carbohydrate esterases (CE), 30 to glycoside hydrolases (GH), 3 to polysaccharide lyases (PL) and 3 to auxiliary activities (AA). Eight sequences are assigned to carbohydrate binding modules (CBM). Table S2 lists the CAZymes identified in the corncob culture: the 30 enzymes are allocated as 5 CEs, 23 GHs, 1 AA and 1 glycoside transferase. Seven sequences are assigned to CBMs. Of the CAZymes identified, 15 are found in both cultures (Table 1): 1 CE, 13 GHs and 1 AA. Six of the GHs correspond to possible cellulases, in agreement with the presence of cellulose in both carbon sources. Table 2 lists the enzymes found solely in the corncob secretome. They correspond mainly to xylanases, which fits to the corncob composition (35% xylan). The enzymes found only in the sugar beet pulp secretome are listed in Table 3. The majority may be grouped as pectinases, again in correspondence to the composition of sugar beet pulp (50% pectin). The results are summarised in Figure 1.

**Table 1.** CAZymes found in the secretome of both corncob and sugar beet pulp cultures.

ID	CAZy family	Putative function	Highest % identity to
evm.model.PPSCF00016.387	CE 16	acetyl esterase	66; *Penicillium oxalicum*
evm.model.PPSCF00016.387	GH 5	endoglucanase	81; *Penicillium oxalicum*
evm.model.PPSCF00061.73	GH 5	endoglucanase	77; *Penicillium oxalicum*
evm.model.PPSCF00095.57	GH 5	endoglucanase	88; *Penicillium brasilianum*
evm.model.PPSCF00015.906	GH 6	cellobiohydrolase	78; *Penicillium oxalicum*
evm.model.PPSCF00019.17	GH 6	cellobiohydrolase	72; *Aspergillus oryzae*
evm.model.PPSCF00016.428	GH 7	cellobiohydrolase	83; *Penicillium decumbens*
evm.model.PPSCF00002.597	GH 17	glucanosyltransferase	86; *Penicillium oxalicum*
evm.model.PPSCF00066.36	GH 18	chitinase	86; *Penicillium oxalicum*
evm.model.PPSCF00061.37	GH 27	alpha-galactosidase^a^	*P. purpurogenum*
evm.model.PPSCF00033.213	GH 31	alpha-glucosidase	84; *Penicillium oxalicum*
evm.model.PPSCF00002.127	GH 35	beta-galactosidase	98; *Penicillium* sp.
evm.model.PPSCF00002.183	GH 47	alpha-mannosidase	86; *Penicillium oxalicum*
evm.model.PPSCF00010.44	GH 131	hypothetical protein	78; *Penicillium oxalicum*
evm.model.PPSCF00016.146	AA 9	cellulose monooxygenase	84; *Penicillium oxalicum*

ID corresponds to the number assigned to the gene in the genome sequence (Mardones et al. 2018). The annotation is based on sequence similarities obtained by Delta BLAST analysis. ^a^Characterised (Morales-Quintana et al. 2017); GenBank N° KP313783.

**Table 2.** CAZymes found only in the corncob secretome.

ID	CAZy family	Putative function	Highest % identity to
evm.model.PPSCF00001.34	CE 1	feruloyl esterase	67; *Penicillium oxalicum*
evm.model.PPSCF00004.579	CE 1	feruloyl esterase	52; *Colletotrichum gloesporoides*
evm.model.PPSCF00035.227	CE 1	feruloyl esterase	74; *Aspergillus oryzae*
evm.model.PPSCF00429.20	CE 3	esterase	58; *Aspergillus fumigatus*
evm.model.PPSCF00062.42	GH 3	beta-xylosidase	*P. purpurogenum^a^*
evm.model.PPSCF00002.263	GH 5	endoglucanase	75; *Penicillium oxalicum*
evm.model.PPSCF00002.458	GH 10	endoxylanase	81; *Penicillium oxalicum*
evm.model.PPSCF00028.25	GH 10	endoxylanase A	*P. purpurogenum^b^*
evm.model.PPSCF00032.82	GH 10	endoxylanase	76; *Penicillium oxalicum*
evm.model.PPSCF0002.743	GH 43	arabinofuranosidase 3	*P. purpurogenum^c^*
evm.model.PPSCF00010.231	GH 43	glycoside hydrolase	77; *Penicillium expansum*
evm.model.PPSCF00015.697	GH 43	ß-xylosidase-arabinofuranosidase	74; *Penicillium oxalicum*
evm.model.PPSCF00015.78	GH 62	arabinofuranosidase	77; *Aspergillus sojae*
evm.model.PPSCF00066.66	GH 78	hypothetical protein	82; *Penicillium oxalicum*
evm.model.PPSCF00002.654	GT 66	oligosaccharyltransferase	88; *Neosartorya fischeri*

ID corresponds to the number assigned to the gene in the genome sequence (Mardones et al. 2018). The proteins have been annotated based on sequence similarities obtained by Delta BLAST. ^a^Biochemically characterised (Faúndez et al. unpublished); GenBank N°KP313787. ^b^Biochemically characterised (Belancic et al. 1995); GenBank N° AAF71268. ^c^Biochemically characterised (Ravanal et al. 2010); GenBank N° FJ906695.

**Table 3.** CAZymes found only in the sugar beet pulp secretome.

ID	CAZy family	Putative function	Highest % identity to
evm.model.PPSCF00026.184	CE 8	pectin methyl esterase	83; *Penicillium oxalicum*
evm.model.PPSCF00020.350	CE 10	pectin acetyl esterase^a^	*P. purpurogenum*
evm.model.PPSCF00014.473	CE 10	carboxyl esterase	55; *Aspergillus kawashii*
evm.model.PPSCF00046.51	CE 12	rhamnogalacturonan acetyl esterase^b^	*P. purpurogenum*
evm.model.PPSCF00048.284	CE 16	carbohydrate esterase	44; *Botryotina fuckeliana*
evm.model.PPSCF00014.422	GH 2	beta-glucuronidase	81; *Penicillium oxalicum*
evm.model.PPSCF00032.82	GH 10	xylanase	76; *Penicillium decumbens*
evm.model.PPSCF00015.932	GH 28	endo-polygalacturonase	84; *Penicillium oxalicum*
evm.model.PPSCF01000.40	GH 28	exo-polygalacturonase	87; *Penicillium oxalicum*
evm.model.PPSCF00048.181	GH 30	endo-beta galactanase	76; *Penicillium oxalicum*
evm.model.PPSCF00015.122	GH 35	hypothetical protein	64; *Pseudogymunoascus pannorum*
evm.model.PPSCF00044.241	GH 35	beta-galactosidase	86; *Penicillium oxalicum*
evm.model.PPSCF00062.7	GH 35	beta-galactosidase	83; *Penicillium oxalicum*
evm.model.PPSCF00035.135	GH 43	exo-beta (1,3) galactanase	78; *Penicillium oxalicum*
evm.model.PPSCF00052.41	GH 43	arabinofuranosidase	79; *Penicillium oxalicum*
evm.model.PPSCF00010.19	GH 51	arabinofuranosidase 2^c^	*P. purpurogenum*
evm.model.PPSCF00024.186	GH 54	arabinofuranosidase 4^d^	*P. purpurogenum*
evm.model.PPSCF00061.89	GH 54	arabinofuranosidase 1^e^	*P. purpurogenum*
evm.model.PPSCF00447.32	GH 75	chitosanase	70; *Penicillium oxalicum*
evm.model.PPSCF00014.488	GH 81	endo-beta (1,3) glucanase	74; *Penicillium oxalicum*
evm.model.PPSCF00035.45	GH 93	exo-arabinanase^f^	*P. purpurogenum*
evm.model.PPSCF00015.181	GH 125	hypothetical protein	82; *Penicillium oxalicum*
evm.model.PPSCF00009.54	PL 1	pectate lyase	85; *Penicillium oxalicum*
evm.model.PPSCF00004.113	PL 4	rhamnogalacturonan lyase	79; *Penicillium oxalicum*
evm.model.PPSCF000062.75	PL 4	rhamnogalacturonan lyase	80; *Penicillium oxalicum*
evm.model.PPSCF0008.77	AA7	FAD dependent oxygenase	70; *Aspergillus flavus*
evm.model.PPSCF00038.120	AA 7	hypothetical protein	76; *Penicillium oxalicum*

ID corresponds to the number assigned to the genes in the genome sequence (Mardones et al. 2018). The proteins have been annotated based on sequenced similarities searches by means of Delta BLAST. ^a^Characterised (Oleas et al. 2017); GenBank N°KP313780. ^b^Characterised (Oleas et al. 2017); GenBank N°KP313786. ^c^Characterised (Fritz et al. 2008); GenBank N° EF490448. ^d^Characterised (Ravanal and Eyzaguirre 2015); GenBank N° AGR66205. ^e^Characterised (De Ioannes et al. 2000); GenBank Nº AAK51551. ^f^Characterised (Mardones et al. 2015); GenBank N° KP313779.

Previous work in our laboratory has analysed the secretome of a sugar beet pulp culture using 2D electrophoresis and mass spectrometry (Navarrete et al. 2012). Thirteen CAZymes were identified, belonging to 10 GH, 1 PL and 1 CE families (Table 4); all of them have their corresponding gene in the *P. purpurogenum* genome, and seven are present in this work’s secretome. It is interesting to note that the strategy used in this work for secretome analysis allows the identification of a higher number of proteins; however, the fact that some enzymes were found only in the 2D electrophoresis analysis suggests that both approaches may be complementary.

**Table 4.** CAZymes found in the sugar beet pulp secretome as determined by 2D electrophoresis and mass spectroscopy by Navarrete et al. (2012), and in this work.

CAZymes identified by (Navarrete et al. 2012)	Genome ID	CAZy family	Present in both cultures	Present only in (Navarrete et al. 2012)
gi|70986018 Cellobiohydrolase	evm.model.PPSCF00015.906	GH 6	+	
gi|115397177 Cellobiohydrolase	evm.model.PPSCF00016.428	GH 7	+	
gi|729650 Endoglucanase	evm.model.PPSCF00001.53	GH 7		+
gi|212538175 Glucan1,4-alpha-glucosidase	evm.model.PPSCF00016.257	GH 15		+
gi|258568902 Glucan 1,3 beta glucosidase	evm.model.PPSCF00002.597	GH 17	+	
gi|238490452 endo-polygalacturonase	evm.model.PPSCF00020.408	GH 28		+
gi|44844271 Beta-galactosidase	evm.model.PPSCF00002.127	GH 35	+	
gi|45826518 Alpha-mannosidase	evm.model.PPSCF00002.183	GH 47	+	
gi|73808014 Exo-beta-1,3-glucanase	evm.model.PPSCF00032.111	GH 55		+
gi|119497763 Pectin lyase^a^	evm.model.PPSCF00015.779	PL 1		+
gi│144228145 arabinofuranosidase^b^	evm.model.PPSCF00010.19	GH 51	+	
gi│13991905 arabinofuranosidase^c^	evm.model.PPSCF00061.89	GH 54	+	
gi│74626767 acetyl xylan esterase II^d^	evm.model.PPSCF00020.380	CE 5		+

Genome ID corresponds to the number assigned to the genes in the genome sequence (Mardones et al. 2018). ^a^Characterised (Perez-Fuentes et al. 2014); GenBank Nº KC751539. ^b^Characterised (Fritz et al. 2008); GenBank Nº EF490448. ^c^Characterised (De Ioannes et al. 2000); GenBank Nº AF367026. ^d^Characterised (Egaña et al. 1996); GenBank Nº AAC39371.

Most work in the field of fungal biodegradation of lignocellulose has been performed with strains of *Trichoderma* and *Aspergillus*. However, in recent years interest in the use of *Penicillium* strains for this purpose has increased (Gusakov and Sinitsyn 2012), and work on the analysis of CAZymes in *Penicillium* secretomes is appearing in the literature. Guais et al. (2008) have analysed the secretome of *Penicillium funiculosum*; a protein cocktail called “RovabioTM Excel” is obtained from culture supernatants of this fungus when grown under industrial process fermentation with cellulose and corn steep liquor as carbon and nitrogen sources. The authors use 2D electrophoresis, 1D electrophoresis and “shotgun” proteomics followed by MS/MS to identify proteins. By means of these complementary methods, they are able to identify more than 50 proteins, including cellulases and hemicellulases; however, a lack of a genome sequence has limited the significance of their analysis. Jami et al. (2010) characterised the extracellular proteome of *Penicillium chrysogenum* grown on glucose; they identify 131 proteins by similarity searches including 18 potentially related to plant cell wall degradation. *Penicillium oxalicum* (formerly *Penicillium decumbens*) has been utilised in China for industrial scale cellulase production. Liu et al. (2013) using a high cellulase-producing mutant of this strain have sequenced its genome and analysed its transcriptome and secretome. The fungus was grown on glucose and on cellulose plus wheat bran, and 21 plant cell-wall degrading enzymes were identified, and its CAZyme composition compared favourably to that of the *T. reesei* secretome. Liao et al. (2014) studied the transcriptome and secretome of a different strain of *P. oxalicum* grown on glucose, xylan, cellulose and xylan plus cellulose. By means of LC-MS/MS, they identified in the secretome a total of 254 proteins, 160 of them hypothetical and the rest mainly putative cellulases and xylanases. Their main finding is that a good correlation exists between the nature of the carbon source and the pattern of enzymes secreted (xylanases in the xylan and cellulases in the cellulose culture). Kaur and Chadha (2015) have grown *Penicillium janthinellum* on rice straw and wheat bran. By means of 2D gels followed by MS, they identified 24 proteins, 16 hypothetical, 6 as GH and 2 without family. Ribeiro et al. (2012) studied the secretome of *Penicillium echinulatum* grown on sugar cane bagasse (untreated and pre-treated) and cellulose as carbon sources using “shotgun” proteomics. They identified 99 proteins, about 90% with predicted functions and the majority (74%) as CAZymes. The substrates used were composed mainly (over 50%) of cellulose, and the enzymes identified were largely directed to cellulose saccharification. From these analyses of the secretome of *Penicillium* species, it is clear that this genus includes a number of species of ample potential for industrial use in the field of lignocellulose biodegradation.

The results obtained in this work show a large number of potentially new CAZymes. In order to fully understand their functionality, a next step requires their biochemical analysis and substrate specificity determination. As Lombard et al. (2014) pointed out, there is a “continuously growing gap between the number of sequences and the number of biochemically or structurally characterised CAZymes”. Therefore, a larger effort will be necessary, not only to close this gap, but also to learn in detail how the lignocellulose polysaccharides are degraded in nature. This knowledge will also be of help to improve the biotechnological applications involving the degradation of these polysaccharides.

## 4. Conclusions

The analysis of the secretome shows that the fungus secretes a high number of CAZymes, and that the pattern of enzyme expression follows the composition of the carbon source used for fungal growth. The results suggest a high potential for the use of *P. purpurogenum* in biotechnological applications related to lignocellulose biodegradation.
