# Supplementary material for: Corncob and sugar beet pulp induce specific sets of lignocellulolytic enzymes in Penicillium purpurogenum
Source: Mycology. 2018 Sep 11;10(2):118–25. doi: 10.1080/21501203.2018.1517830 (PMC6493289; doi:10.1080/21501203.2018.1517830)
Supplement: Supplemental Material [file TMYC_A_1517830_SM1535.zip › Table_S1.docx]

**Table S1**

CAZymes present in the sugar beet pulp secretome.

| **Protein Id** | **CAZy Family^+^** | **e-value*** |
| --- | --- | --- |
| **evm.model.PPSCF00002.127** | GH35 | 2.4e-94 |
| **evm.model.PPSCF00002.183** | GH47 | 7.7e-137 |
| **evm.model.PPSCF00002.597** | GH17 | 1.0e-40 |
| **evm.model.PPSCF00004.113** | PL4 | 2.5e-95 |
| **evm.model.PPSCF00008.77** | AA7 | 8.2e-48 |
| **evm.model.PPSCF00009.54** | PL1 | 8.3e-55 |
| **evm.model.PPSCF00010.19** | GH51 | 4.5e-58 |
| **evm.model.PPSCF00010.44** | GH131 | 2.7e-98 |
| **evm.model.PPSCF00014.422** | GH2 | 5.8e-104 |
| **evm.model.PPSCF00014.473** | CE10 | 1.7e-26 |
| **evm.model.PPSCF00014.488** | GH81 | 2.9e-229 |
| **evm.model.PPSCF00015.122** | GH35 | 8.4e-94 |
| **evm.model.PPSCF00015.181** | GH125 | 2.9e-165 |
| **evm.model.PPSCF00015.906** | CBM1 | 3.1e-12 |
| **evm.model.PPSCF00015.906** | GH6 | 9.3e-95 |
| **evm.model.PPSCF00015.932** | GH28 | 9.6e-73 |
| **evm.model.PPSCF00016.146** | AA9 | 1.3e-73 |
| **evm.model.PPSCF00016.387** | CE16 | 7.3e-79 |
| **evm.model.PPSCF00016.387** | GH5 | 4.0e-35 |
| **evm.model.PPSCF00016.428** | GH7 | 1.2e-197 |
| **evm.model.PPSCF00019.17** | GH6 | 1.1e-93 |
| **evm.model.PPSCF00020.350** | CE10 | 4.3e-28 |
| **evm.model.PPSCF00024.186** | GH54 | 3.4e-155 |
| **evm.model.PPSCF00024.186** | CBM42 | 1.4e-53 |
| **evm.model.PPSCF00026.184** | CE8 | 1.2e-58 |
| **evm.model.PPSCF00032.82** | GH10 | 1.4e-99 |
| **evm.model.PPSCF00032.82** | CBM1 | 2.9e-15 |
| **evm.model.PPSCF00033.213** | GH31 | 1.7e-150 |
| **evm.model.PPSCF00035.135** | GH43 | 9.2e-30 |
| **evm.model.PPSCF00035.135** | CBM35 | 9.5e-17 |
| **evm.model.PPSCF00035.45** | GH93 | 2.5e-119 |
| **evm.model.PPSCF00038.120** | AA7 | 3.3e-49 |
| **evm.model.PPSCF00044.241** | GH35 | 3.4e-81 |
| **evm.model.PPSCF00046.51** | CE12 | 2.1e-47 |
| **evm.model.PPSCF00048.181** | GH30 | 4.1e-33 |
| **evm.model.PPSCF00048.284** | CE16 | 2.0e-78 |
| **evm.model.PPSCF00052.41** | GH43 | 3.4e-42 |
| **evm.model.PPSCF00061.37** | GH27 | 3.1e-33 |
| **evm.model.PPSCF00061.73** | CBM1 | 2.8e-11 |
| **evm.model.PPSCF00061.73** | GH5 | 1.4e-31 |
| **evm.model.PPSCF00061.73** | CBM46 | 3.2e-16 |
| **evm.model.PPSCF00061.89** | GH54 | 9.2e-160 |
| **evm.model.PPSCF00061.89** | CBM42 | 6.9e-61 |
| **evm.model.PPSCF00062.7** | GH35 | 4.0e-82 |
| **evm.model.PPSCF00062.7** | CBM67 | 6.5e-05 |
| **evm.model.PPSCF00062.75** | PL4 | 1.2e-173 |
| **evm.model.PPSCF00066.36** | GH18 | 2.6e-55 |
| **evm.model.PPSCF00095.57** | GH5 | 5.0e-41 |
| **evm.model.PPSCF00447.32** | GH75 | 2.8e-68 |
| **evm.model.PPSCF01000.40** | GH28 | 1.6e-69 |

ID corresponds to the number assigned to the gene in the genome sequence ([Mardones et al. 2018](#_ENREF_1))

^+^CAZy family assigned by dbCAN

*Confidence value assigned by dbCAN

Mardones, W., Di Genova, A., Cortés, M.P., Travisany, D., Maass, A., Eyzaguirre, J., 2018. The genome sequence of the soft-rot fungus Penicillium purpurogenum reveals a high gene dosage for lignocellulolytic enzymes. Mycology, 1-11.
