# Supplementary material for: Corncob and sugar beet pulp induce specific sets of lignocellulolytic enzymes in Penicillium purpurogenum
Source: Mycology. 2018 Sep 11;10(2):118–25. doi: 10.1080/21501203.2018.1517830 (PMC6493289; doi:10.1080/21501203.2018.1517830)
Supplement: Supplemental Material [file TMYC_A_1517830_SM1535.zip › Table_S2.docx]

**Table S2**

CAZymes present in the corncob secretome

| **Protein Id** | **CAZy Family^+^** | **e-value*** |
| --- | --- | --- |
| **evm.model.PPSCF00001.34** | CBM1 | 4.7e-12 |
| **evm.model.PPSCF00001.34** | CE1 | 3.1e-25 |
| **evm.model.PPSCF00002.127** | GH35 | 2.4e-94 |
| **evm.model.PPSCF00002.183** | GH47 | 7.7e-137 |
| **evm.model.PPSCF00002.263** | CBM1 | 9.5e-13 |
| **evm.model.PPSCF00002.263** | GH5 | 2.2e-38 |
| **evm.model.PPSCF00002.458** | GH10 | 1.0e-87 |
| **evm.model.PPSCF00002.597** | GH17 | 1.0e-40 |
| **evm.model.PPSCF00002.654** | GT66 | 2.4e-194 |
| **evm.model.PPSCF00002.743** | GH43 | 1.1e-53 |
| **evm.model.PPSCF00002.743** | CBM6 | 1.9e-38 |
| **evm.model.PPSCF00004.579** | CE1 | 6.9e-07 |
| **evm.model.PPSCF00010.231** | GH43 | 2.9e-42 |
| **evm.model.PPSCF00010.44** | GH131 | 2.7e-98 |
| **evm.model.PPSCF00015.697** | GH43 | 2.8e-45 |
| **evm.model.PPSCF00015.78** | GH62 | 1.0e-134 |
| **evm.model.PPSCF00015.906** | CBM1 | 3.1e-12 |
| **evm.model.PPSCF00015.906** | GH6 | 9.3e-95 |
| **evm.model.PPSCF00016.146** | AA9 | 1.3e-73 |
| **evm.model.PPSCF00016.387** | CE16 | 7.3e-79 |
| **evm.model.PPSCF00016.387** | GH5 | 4.0e-35 |
| **evm.model.PPSCF00016.428** | GH7 | 1.2e-197 |
| **evm.model.PPSCF00019.17** | GH6 | 1.1e-93 |
| **evm.model.PPSCF00028.25** | GH10 | 2.4e-105 |
| **evm.model.PPSCF00032.82** | GH10 | 1.4e-99 |
| **evm.model.PPSCF00032.82** | CBM1 | 2.9e-15 |
| **evm.model.PPSCF00033.213** | GH31 | 1.7e-150 |
| **evm.model.PPSCF00035.227** | CE1 | 9.9e-05 |
| **evm.model.PPSCF00061.37** | GH27 | 3.1e-33 |
| **evm.model.PPSCF00061.73** | CBM1 | 2.8e-11 |
| **evm.model.PPSCF00061.73** | GH5 | 1.4e-31 |
| **evm.model.PPSCF00061.73** | CBM46 | 3.2e-16 |
| **evm.model.PPSCF00062.42** | GH3 | 2.5e-52 |
| **evm.model.PPSCF00066.36** | GH18 | 2.6e-55 |
| **evm.model.PPSCF00066.66** | GH78 | 1.3e-07 |
| **evm.model.PPSCF00095.57** | GH5 | 5.0e-41 |
| **evm.model.PPSCF00429.20** | CE3 | 1.4e-42 |

ID: corresponds to the number assigned to the gene in the genome sequence ([Mardones et al. 2018](#_ENREF_1))

^+^CAZy famiy assigned by dbCAN

*Confidence value assigned by dbCAN
