# Supplementary figures and images for: Corncob and sugar beet pulp induce specific sets of lignocellulolytic enzymes in Penicillium purpurogenum
Source: Mycology. 2018 Sep 11;10(2):118–25. doi: 10.1080/21501203.2018.1517830 (PMC6493289; doi:10.1080/21501203.2018.1517830)

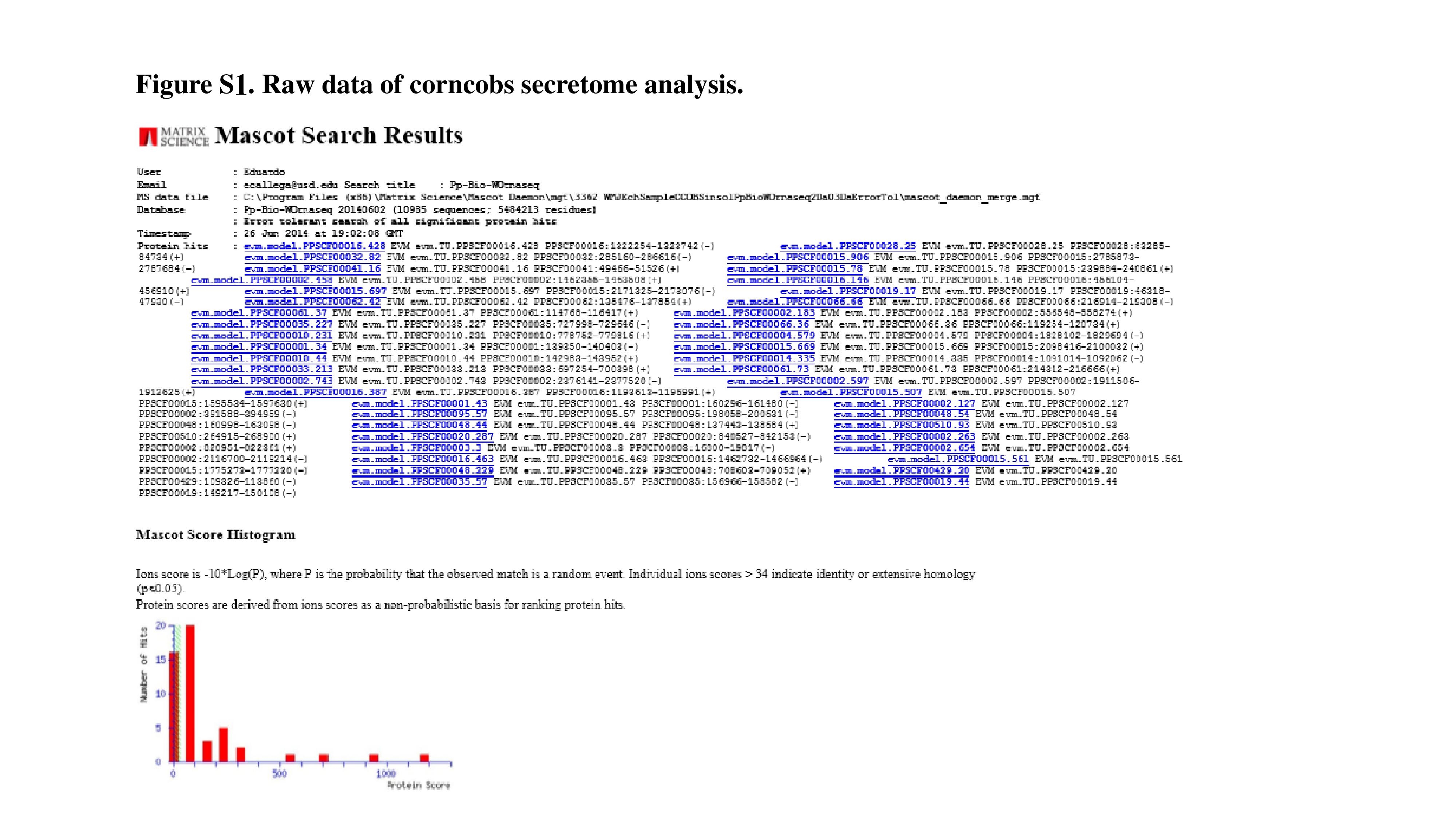

Supplement: Supplemental Material [file TMYC_A_1517830_SM1535.zip › Figure_S1.jpg]

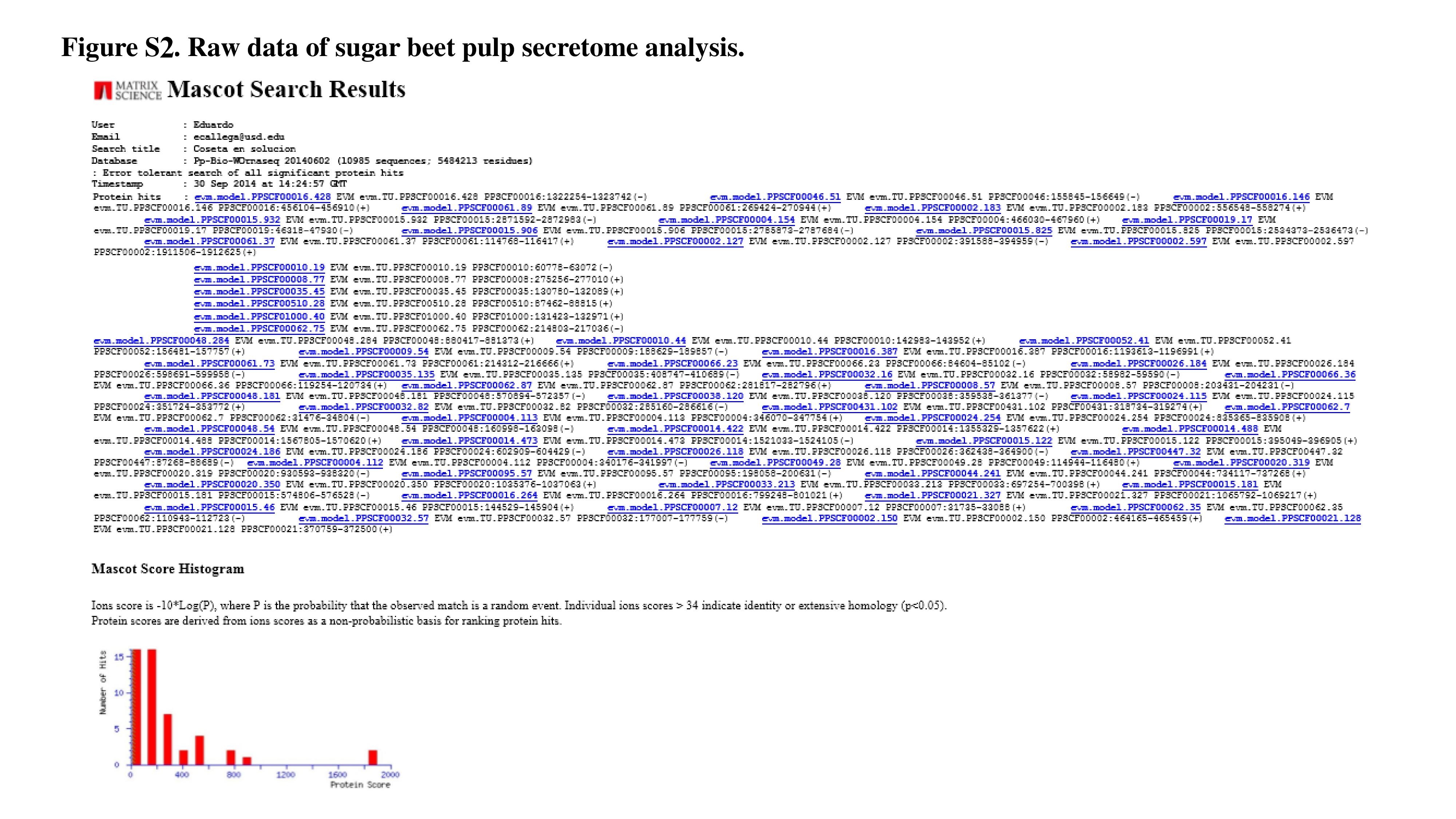

Supplement: Supplemental Material [file TMYC_A_1517830_SM1535.zip › Figure_S2.jpg]
